# Streptococcal M1 protein induces hyporesponsiveness and cytokine release from human arteries in a fibrinogen-dependent manner: a translational study

**DOI:** 10.1186/s13049-018-0530-1

**Published:** 2018-07-24

**Authors:** Viveka Björck, Lisa I. Påhlman, Johan Törnebrant, Mikael Bodelsson

**Affiliations:** 1 Department of Clinical Sciences Lund, Lund University, Skane University Hospital, Anaesthesiology and intensive care, SE-221 85 Lund, Sweden; 2 Department of Clinical Sciences Lund, Lund University, Skane University Hospital, Infection medicine, SE-221 85 Lund, Sweden

**Keywords:** Sepsis, M1 protein, Hypotension, Cytokine, Interleukin, Fibrinogen, Streptococcus, Artery

## Abstract

**Background:**

*Streptococcus pyogenes* is a Gram positive bacterial species commonly involved in sepsis. Invasive strains express virulence factors such as the M1 protein. M1 protein forms complexes with fibrinogen leading to a cytokine storm in plasma contributing to the development of septic shock and organ failure. In experimental animals M1 protein causes vascular nitric oxide production and hyporesponsiveness to pressors, but it is not known whether it affects the human vascular wall.

**Methods:**

Human omental arteries obtained during surgery were incubated in vitro with M1 protein or lipopolysaccharide (LPS) as positive control, with or without plasma. After 48 h, contractile response to noradrenaline was measured, and levels of nitrite/nitrate and the cytokines interleukin (IL)-1β, IL-6, IL-8, IL-10, and tumor necrosis factor (TNF)-α in the incubation medium were measured. A second set of arteries were incubated with or without main components of plasma (immunoglobulin G, albumin or fibrinogen), in the presence of M1 protein followed by cytokine measurement.

**Results:**

Artery segments incubated with M1 protein and plasma contracted weaker in response to noradrenaline, and levels of IL-6 and IL-8 were significantly higher compared to after incubation with M1 protein alone. Incubation with M1 protein and fibrinogen resulted in elevated levels of IL-6 and IL-8, while incubation with M1 protein and albumin or immunoglobulin G did not affect the levels. Neither any of the other cytokines nor nitrite/nitrate was detected in the medium in any of the incubation conditions.

**Conclusions:**

The study shows that M1 protein of *Streptococcus pyogenes* has a direct effect on the human vascular wall in the presence of plasma, demonstrated both as a diminished contractile response to noradrenaline and increased cytokine production. The effect of plasma was attributed to fibrinogen. The findings suggest that M1 protein contributes to the development of septic shock through impairment of the contractility of the vascular wall.

## Background

Sepsis is a devastating condition in which a dysregulated host response to an infection may lead to multiple organ failure. The mortality in sepsis and septic shock is high, at least 25% [1], and early diagnosis and adequate treatment, such as correct antibiotic therapy and support of vital functions, is important to improve survival [2].

The Gram-positive bacterium *Streptococcus pyogenes* is one of the pathogens causing severe sepsis*.* It possesses a group of virulence factors, M proteins [3], some of which stimulate immune cells leading to a cytokine storm and sepsis*,* resulting in tissue damage, disseminated intravascular coagulation and organ dysfunction [4]. M proteins are present on the surface of Group A, C and G streptococci, but can also be found solubilized in plasma. They comprise of over 200 serotypes determined by gene type, and M protein serotyping is used as an epidemiological marker to identify streptococcal isolates [5, 6]. M1 protein (coded by the gene *Emm1*), is one of the most frequently found types expressed by streptococcal Group A isolates from patients presenting with invasive forms of streptococcal infections such as toxic shock syndrome and necrotizing fasciitis [7–9].

Adhesion of bacteria to mucous or dermal membranes is an important first step in establishing an invasive infection, and M proteins are involved in this process by binding to fibronectin and glycosaminoglycans [5]. When bacteremia occurs, a sequence of events follows, for example M1 protein forms complexes with fibrinogen, which cause neutrophils to degranulate and release heparin-binding protein (HBP) [10]. HBP has been claimed to be involved in vascular leakage, one of the main characteristics in sepsis leading to edema, hypovolemia and subsequent decreased oxygen delivery to organs [11]. M1 protein also produces a direct vascular effect. Rat and mouse aorta segments incubated with M1 protein shows a lowered constrictive response to the α-adrenoceptor agonist, phenylephrine, a vasoplegic effect mediated via an M1 protein/Toll-like receptor (TLR)4 interaction [12].

It has been demonstrated that monocytes stimulated with M1 protein in vitro release interleukin (IL)-6 due to an effect of M1 protein on TLR2-receptor [13]. IL-6 is involved in capillary leakage via disruption of VE-cadherin between endothelial cells [14]. This suggests several intertwined pathways contributing to the pathophysiology of streptococcal sepsis. The implications of these findings for human sepsis, including the failing circulation, is unknown, which hampers development of specific therapy. In the present study we therefore wanted to investigate the inflammatory effect of M1 protein on human vasculature.

## Methods

### Human arteries

The project was approved by the Institutional Review Board of the Regional Ethics Committee in Lund, Sweden (LU 18–93 and Dnr 2012/148). After written informed consent a piece of *omentum majus* obtained from ten patients undergoing abdominal surgery for ovarian or uterine cancer. Median age was 60 years (range 46–81). Omental arteries were dissected free from fat and connective tissues and cut into 2–4 mm long segments. Segments were carefully washed in Dulbecco’s modified Eagle’s medium without phenol red (Gibco), to remove blood remnants. Five mL venous blood from healthy donors was drawn into an EDTA coated plastic vacuette and plasma was collected after centrifugation at 1000 × g for 3 min.

### M1 protein

M1 protein was obtained from the isogenic mutant MC25 strain, derived from the AP1 strain (*S. pyogenes* strain from the World Health Organization Collaborating Centre for References and Research on Streptococci, Institute of Hygiene and Epidemiology, Prague, Czech Republic). This M1 protein lacks the membrane-spanning region and secretes a soluble form that was purified as previously described [15]. In short, the supernatant from an MC25 culture was collected, and proteins were precipitated with ammonium sulfate, dissolved in phosphate-buffered saline solution, and purified on fibrinogen-coupled Sepharose. The purity of M1 protein was confirmed by SDS-PAGE analysis. The M1 protein preparation was analyzed for LPS and the LPS was removed using Endotrap from Hyglos GmbH (Bernried am Starnberger See, Germany). The LPS levels after purification was 0.08 EU μg^− 1^ M1.

### Incubations

Arterial segments were incubated at 37 °C in Dulbecco’s modified Eagle’s medium without phenol red (Gibco) in the presence of L-arginine (1 mM), penicillin (2000 U mL^− 1^) and streptomycin (0.2 mg mL^− 1^, all from Sigma-Aldrich), aerated by 8% CO_2_ in O_2_ for 5 min. Pilot experiments demonstrated that incubation with LPS for 48 h resulted in a marked and consistent inflammatory response judged by cytokine release and decreased contractile response to noradrenaline compared to 24 h. Compared to 48 h no further change was registered at 72 h. Thus, in all subsequent experiments the incubation time was 48 h. In the first set of experiments, the following compounds were added: *(a)* control without additives, *(b)* control with 10% plasma, *(c)* LPS (50 EU mL^− 1^, from Sigma-Aldrich), *(d)* LPS 50 EU mL^− 1^ with 10% plasma, *(e)* M1 protein (1 or 10 μg mL^− 1^), *(f)* M1 protein (1 or 10 μg mL^− 1^) with 10% plasma. Artery segments and plasma were obtained from the same patient. In the second set of experiments, the following compounds were added: *(a)* control without additives, *(b)* controls with IgG (1.1 mg mL^− 1^, Octapharma), albumin (4 mg mL^− 1^, Octapharma) or fibrinogen (0.3 mg mL^− 1^, Sigma-Aldrich), respectively, which corresponds to 10% of normal plasma concentrations, *(c)* LPS 50 EU mL^− 1^, *(d)* LPS 50 EU mL^− 1^ with Immunoglobulin G (IgG), albumin or fibrinogen, respectively, *(e)* M1 protein (1 μg mL^− 1^), *(f)* M1 protein (1 μg mL^− 1^) with IgG, albumin or fibrinogen, respectively.

After incubation the artery segments were removed and weighed, and the incubation medium was centrifuged at 5000×g for 5 min. The supernatant was removed and kept at -20 °C until subsequent measurement of cytokine and nitrite/nitrate levels (see below).

### Measurement of smooth muscle force

After incubation, the segments were thread onto two L-shaped hooks in 2-ml tissue baths containing Krebs-Ringer solution (composition in mM: Na^+^ 143, K^+^ 4.6, Cl^−^ 126, Ca^2+^ 2.5, HCO_3_^−^ 25, Mg^2+^ 0.79, SO_4_^2+^ 0.79, H_2_PO_4_^−^ 1.2, glucose 5.5 and ethylene diamine tetra-acetic acid, EDTA, 0.024) aerated with 8% CO_2_ in O_2_ and maintained at 37 °C. One of the hooks was attached to a Grass FTO3C force-displacement transducer for measurement of isometric force. The force was recorded on a Grass polygraph model 7b (Grass Instrument Corp., Quincy, MA, USA). The artery segments were equilibrated for 1 h during which the final pretension was adjusted to 4–8 mN [16]. Potassium chloride (83 mM) was then added and a resulting smooth muscle contraction confirmed viability. After wash-out, noradrenaline (NA, 10^− 10^ – 10^− 4^ M) was added cumulatively in 0.5 _10_log units. The resulting contraction was registered and concentration-response curves were constructed.

### Cytometric bead array

Concentrations of IL-1β, IL-6, IL-8, IL-10, and tumor necrosis factor (TNF)-α in the incubation medium were measured with cytometric bead array (CBA) enhanced sensitivity flex set (BD Biosciences, San Jose, CA) using fluorescence-activated cell sorting (FACS Verse, BD).

### Measurements of nitrite/nitrate production in human arteries

NO is rapidly oxidized to nitrite and nitrate, and therefore levels of these substances can be used as an indicator of NO production. Nitrite/nitrate was measured as previously described [12].

### Analysis of data

Concentration-response curves for noradrenaline-induced contraction was characterized by the maximum response, the negative base 10 logarithm of the noradrenaline concentration eliciting half maximum response (pEC_50_) calculated using linear interpolation, and area under the curve between concentrations 10^− 8^ and 10^− 5^ M. Values obtained were compared using one way repeated measures analysis of variance (RM ANOVA). Two way RM ANOVA was used for the statistical analysis of effects of plasma on the accumulation of IL-6 and IL-8. To analyze the effect of IgG, albumin and fibrinogen, one way RM ANOVA was used. *P <* 0.05 was considered to indicate a statistically significant difference. Values are given as means (SE) and the number of experiments equals the number of patients providing vascular tissue and is given as *n.*

## Results

### Measurement of smooth muscle force

**Fig. 1:**
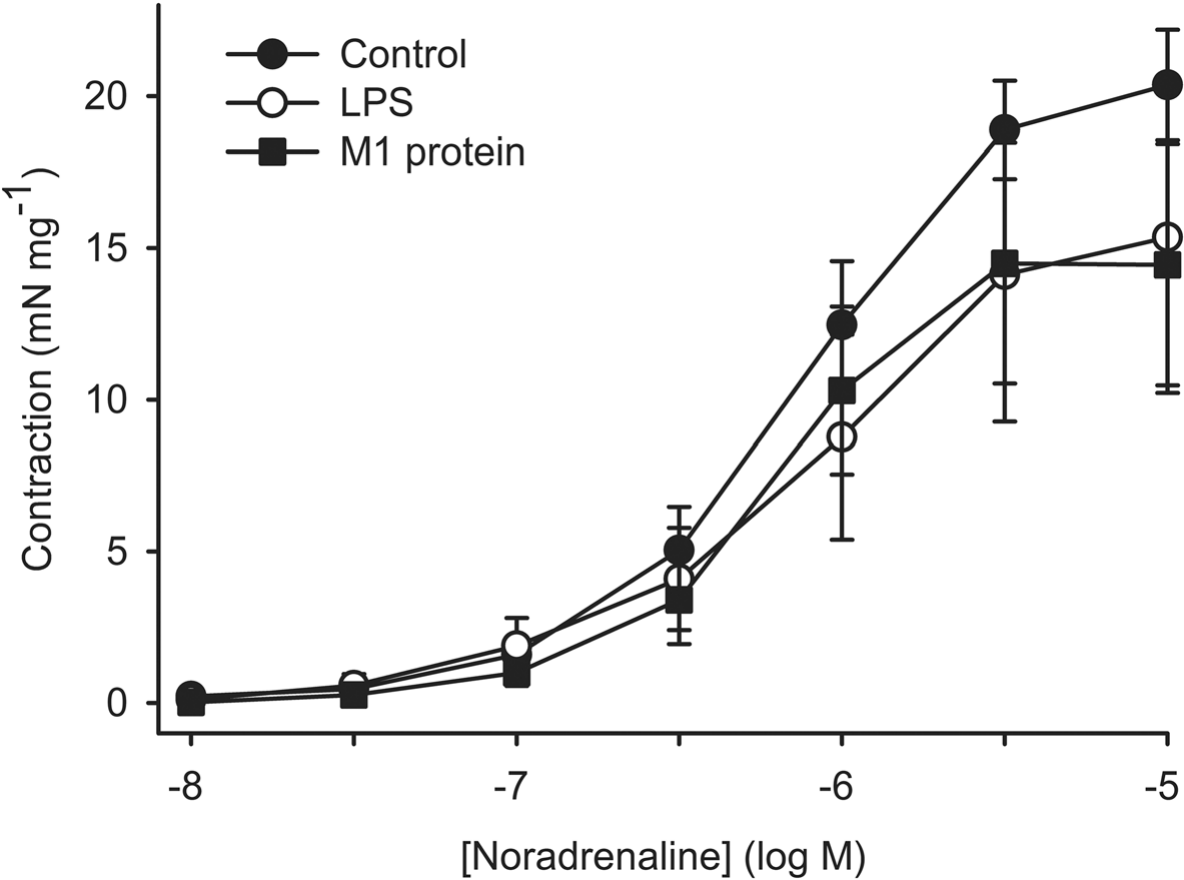
Contraction induced by noradrenaline in human omental arteries incubated without plasma in the presence or absence of LPS (50 EU mL-1) or M1 protein (1 μg mL-1). The concentration-response curve after incubation with LPS or M1 protein did not statistically significantly differ from control regarding maximum response, noradrenaline concentration required to elicit half maximum response or area under the curve. One way repeated measurement ANOVA. Values are means ± SE (*n* = 5)

**Fig. 2:**
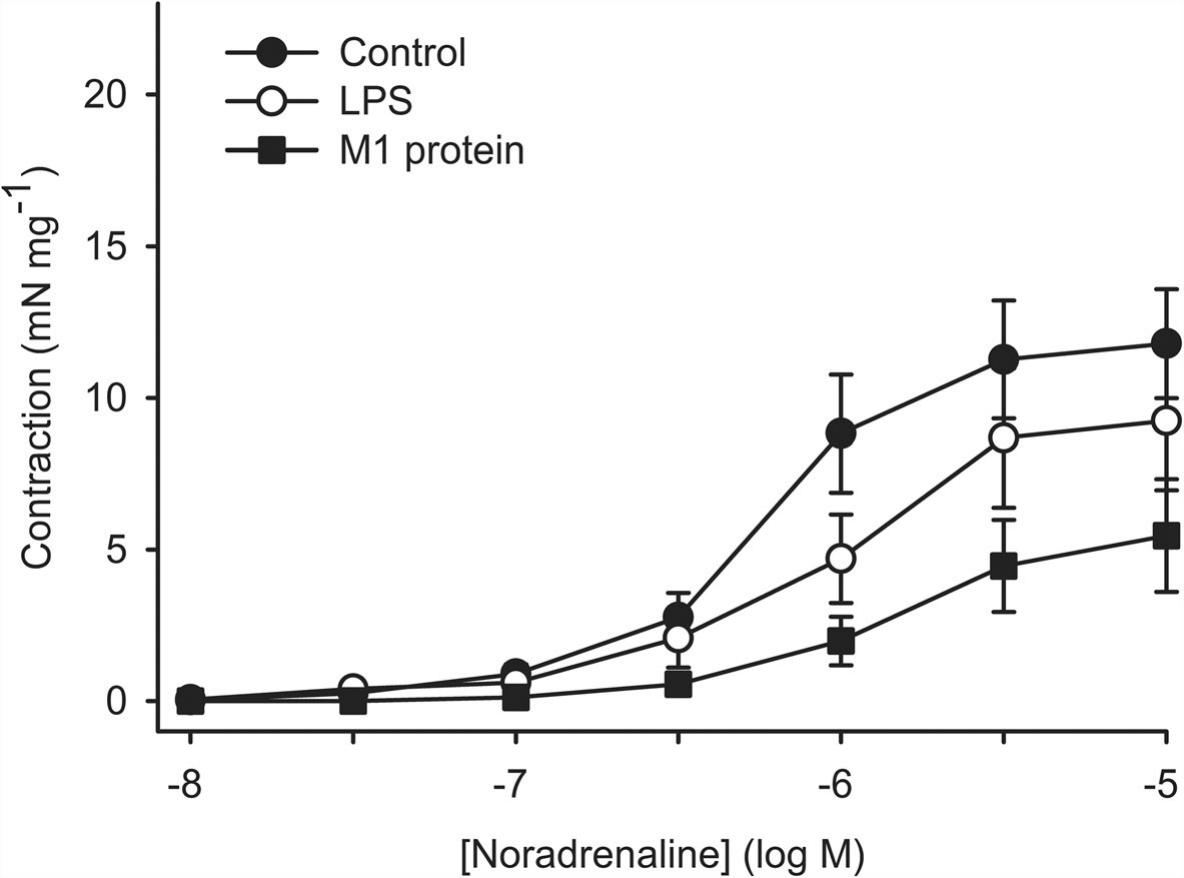
Contraction induced by noradrenaline in human omental arteries incubated with plasma (10%) in the presence or absence of LPS (50 EU mL-1) or M1 protein (1 μg mL-1). The concentration-response curve to noradrenaline was shifted to the right after incubation with M1 protein reflected by an increase in noradrenaline concentration required to elicit half maximum response (*P* = 0.031). The area under the curve was significant smaller (*P* = 0.044) after incubation with M1 protein. The concentration-response curve after incubation with LPS did not statistically significantly differ from control. One way repeated measurement ANOVA. Values are means ± SE (*n* = 5)

Noradrenaline induced a concentration-dependent contraction in the artery segments. After incubation for 48 h with LPS or M1 protein in the absence of plasma, the concentration-response curve for noradrenaline did not statistically significantly differ from control segments incubated in medium only (Fig. 1). In the presence of plasma during incubation with M1 protein the concentration-response curve for noradrenaline was shifted to the right compared to control segments incubated with plasma alone (pEC_50_, 6.20 (0.12) and 5.67 (0.14) after incubation with and without M1 protein, respectively, *P* = 0.031), which indicates that higher concentrations of noradrenalin is needed to reach the same levels of contraction as in the controls (Fig. 2). Furthermore, the area under the noradrenaline-response curve was significantly smaller after incubation with M1 protein and plasma compared to control with plasma alone (13 (3.7) and 36 (6.2) arbitrary units after incubation with and without M1 protein, respectively, *P* = 0.044, Fig. 2). The concentration-response curve after incubation with LPS and plasma did not statistically significantly differ from control incubated with plasma alone.

### Accumulation of cytokines and nitrite/nitrate

**Fig. 3:**
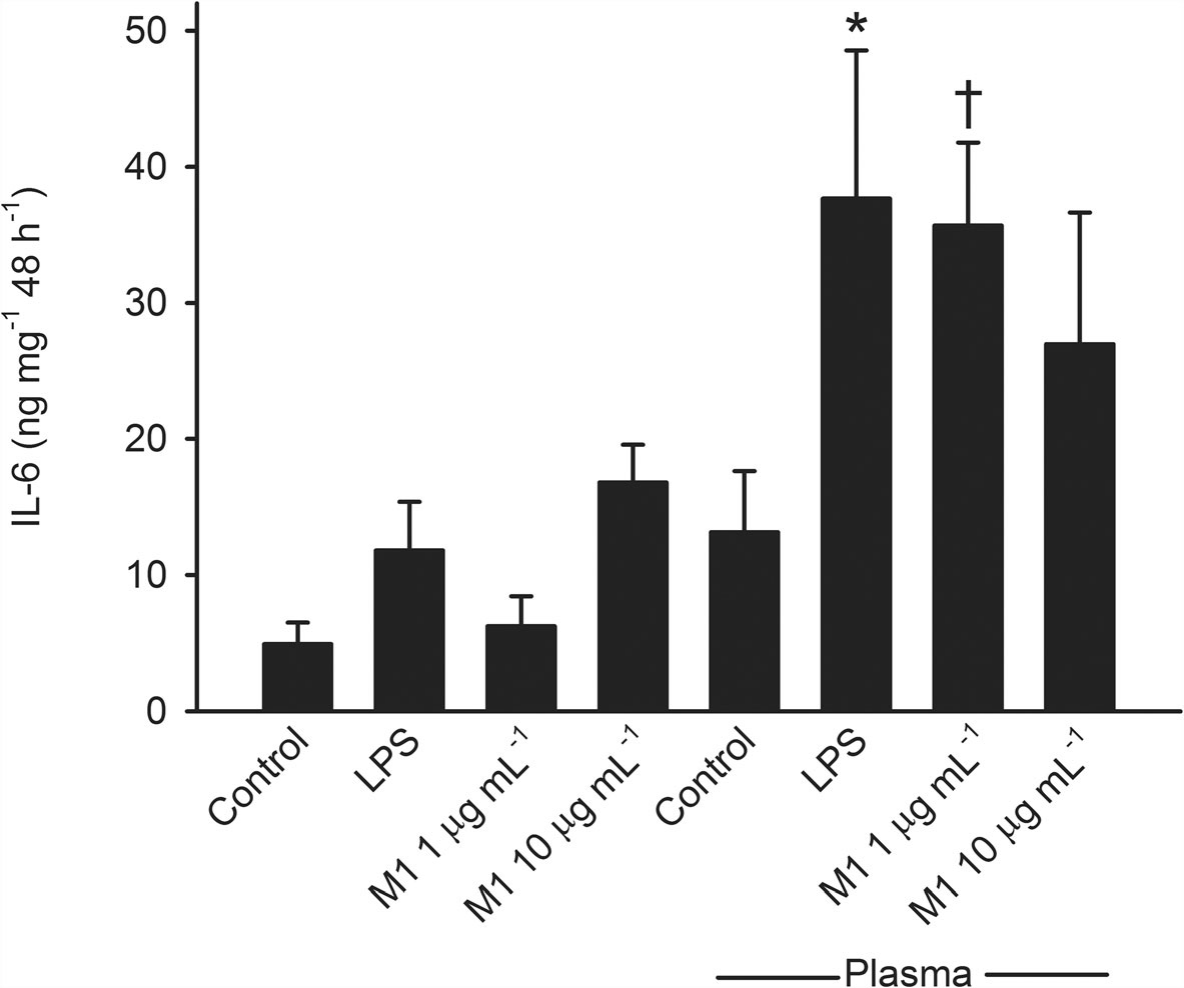
Interleukin (IL)-6 accumulation after incubation of human omental arteries with and without plasma (10%) in the presence of M1 protein and LPS (50 EU mL-1)**.** The presence of plasma caused a statistically significantly greater IL-6 accumulation induced by LPS (*, *P =* 0.002) and M1 protein at 1 μg mL^− 1^ (†, *P <* 0.001). Two way repeated measurement ANOVA for factors plasma and incubation. Values are means ± SE (*n* = 6)

**Fig. 4:**
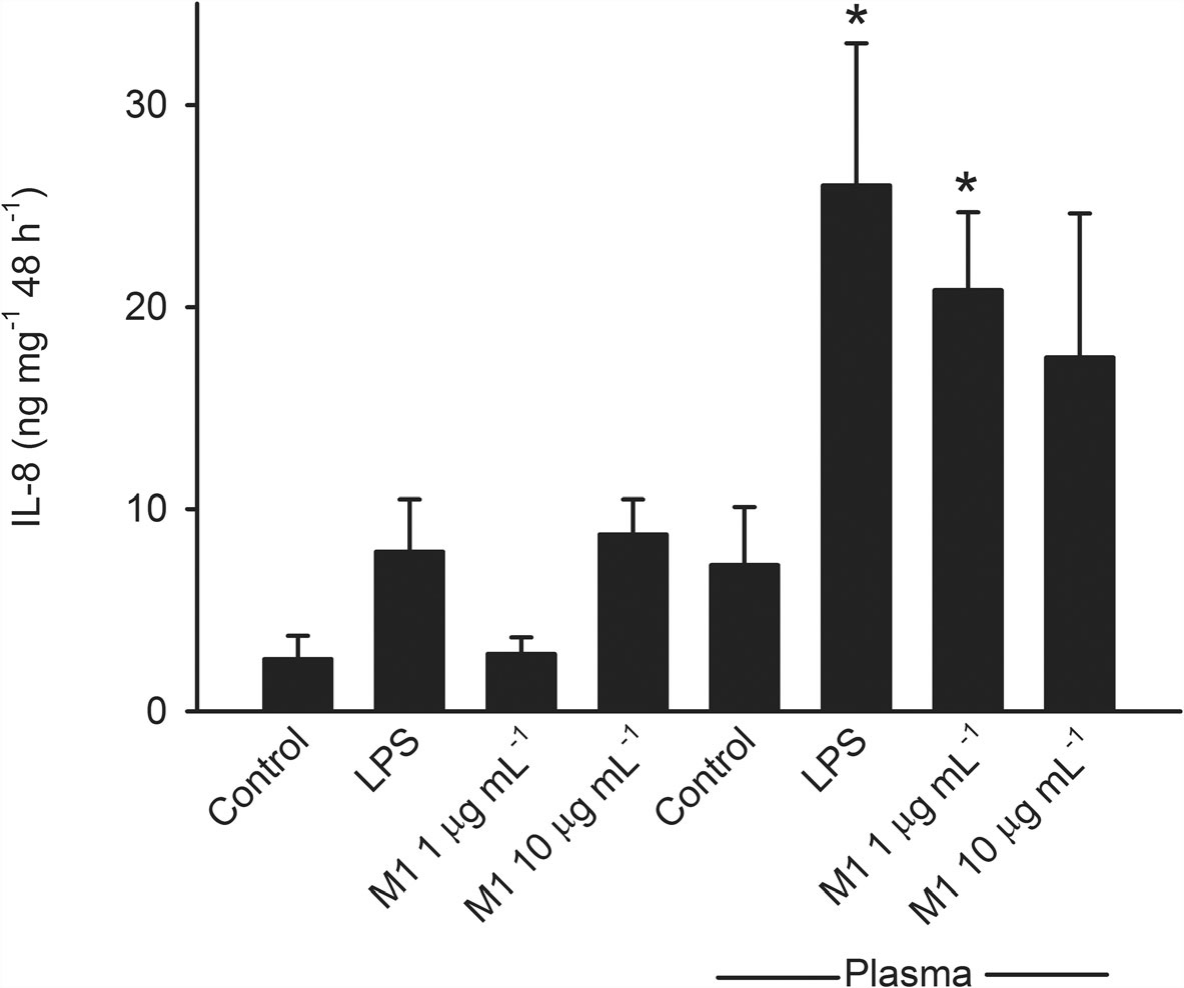
Interleukin (IL)-8 accumulation after incubation of human omental arteries with and without plasma in the presence of M1 protein and LPS (50 EU mL-1**).** The presence of plasma caused a statistically significantly greater IL-8 accumulation induced by LPS (*, *P =* 0.002) and M1 protein at 1 μg mL^− 1^ (*, *P =* 0.002). Two way repeated measurement ANOVA for factors plasma and incubation. Values are means ± SE (*n* = 6)

Incubation of artery segments with M1 protein or LPS for 48 h in the absence of plasma caused only small and statistically non-significant changes in IL-6 and IL-8 accumulation (Figs. 3 and 4). Incubation in the presence of plasma statistically significantly increased IL-6 and IL-8 accumulation induced by M1 protein at 1 μg mL^− 1^ (*P* < 0.001 and *P* = 0.002 for IL-6 and IL-8, respectively) as well as LPS (*P* = 0.002 for both IL-6 and IL-8) compared to incubation in the absence of plasma (Figs. 3 and 4).

**Fig. 5:**
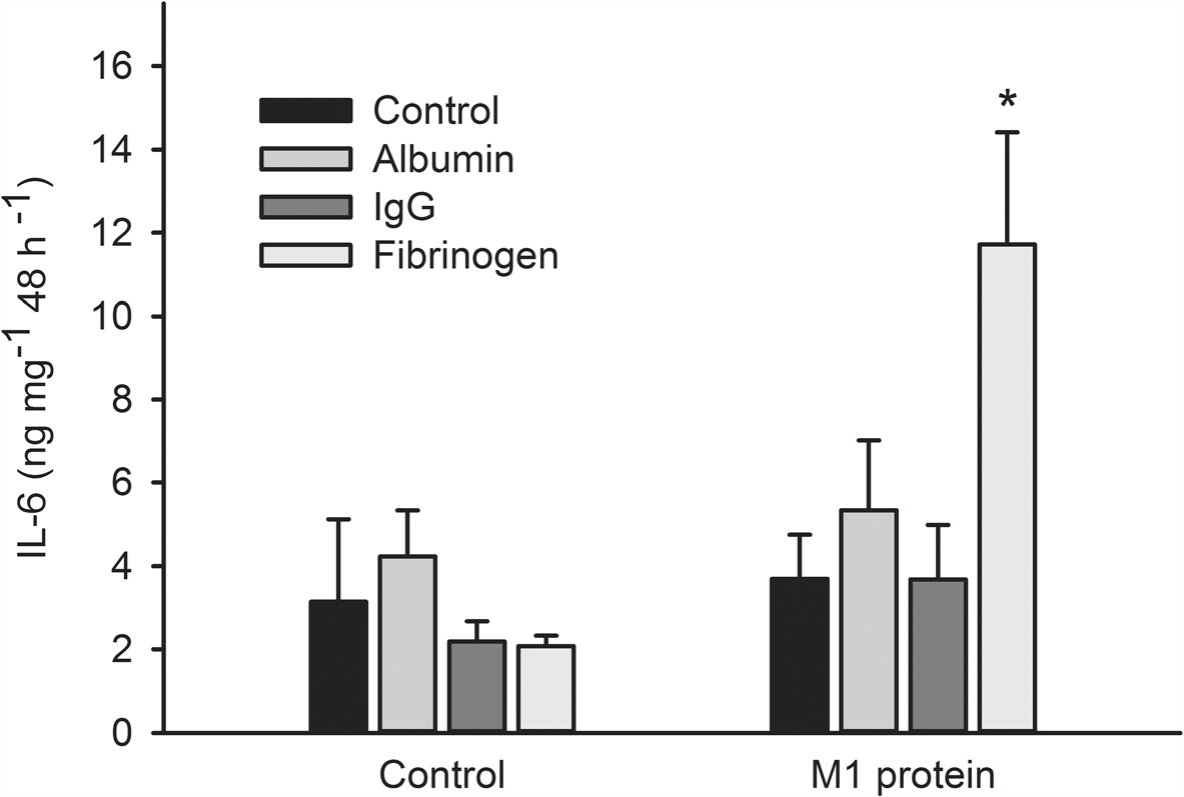
Interleukin (IL)-6 accumulation after incubation of human omental arteries with and without albumin, immunoglobin (Ig) G or fibrinogen in the absence or presence of M1 protein. Incubation in the presence of fibrinogen significantly enhanced the IL-6 accumulation induced by M1 protein (*, *P* = 0.003) while albumin and immunoglobulin G did not affect it. One way repeated measurement ANOVA. Values are means ± SE (*n* = 5). Concentrations were: M1 protein, 1 μg mL^− 1^; albumin, 4 mg mL^− 1^; Ig G, 1.1 mg mL^− 1^; fibrinogen, 0.3 mg mL^− 1^

**Fig. 6:**
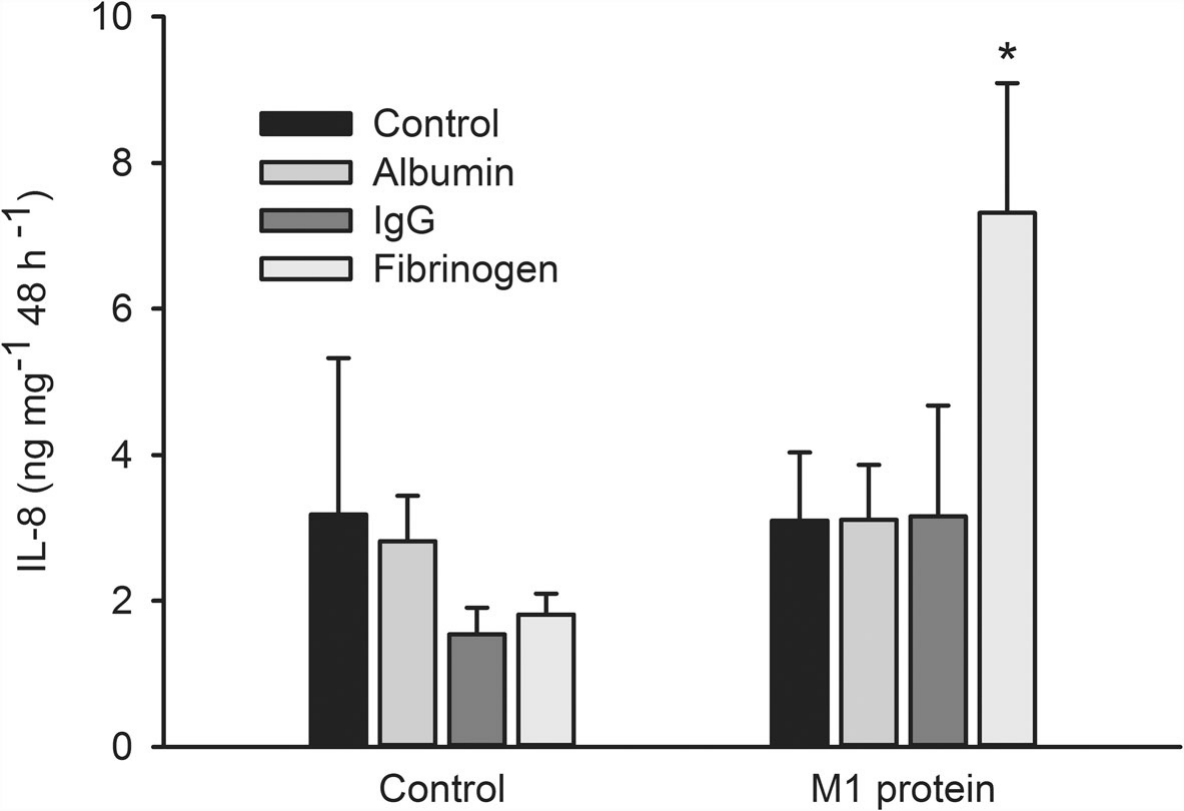
Interleukin (IL)-8 accumulation after incubation of human omental arteries with and without albumin, immunoglobin (Ig) G or fibrinogen in the absence or presence of M1 protein. Incubation in the presence of fibrinogen significantly enhanced the IL-8 accumulation induced by M1 protein (*, *P* = 0.048) while albumin and immunoglobulin G did not affect it. One way repeated measurement ANOVA. Values are means ± SE (*n* = 5). Concentrations were: M1 protein, 1 μg mL^− 1^; albumin, 4 mg mL^− 1^; Ig G, 1.1 mg mL^− 1^; fibrinogen, 0.3 mg mL^− 1^

Further experiments showed that accumulation of IL-6 and IL-8 after incubation of arterial segments with M1 protein was significantly greater in the presence of fibrinogen (*P* = 0.003 and *P* = 0.048 for IL-6 and IL-8, respectively), while albumin and IgG had no effect (Figs. 5 and 6). IL-6 and IL-8 accumulation was significantly greater after incubation with LPS in the presence of albumin (*P* < 0.001 for both IL-6 and IL-8) while fibrinogen and IgG had no effect (not shown). Accumulation of IL-1β, IL-10, TNF-α and nitrite/nitrate was not affected by M1 protein or LPS regardless of presence or absence of plasma, albumin, IgG or fibrinogen.

## Discussion

In the present study we have shown that human arteries, incubated with the streptococcal virulence factor M1 protein in vitro, have a reduced contractile smooth muscle response to noradrenaline, the most frequently used vasoconstrictor in critically ill patients. This finding corresponds to the well-known clinical observation that patients with septic shock do not respond to vasoconstrictors to the same extent as patients without sepsis [1, 17]. The reasons for this seems to be multifactorial, but established mechanisms include a sepsis-induced increase nitric oxide (NO) production by endothelial and inducible NO synthase (NOS) as well as generation of prostacyclin by cyclooxygenase 2 [18, 19]. We have previously shown that release of NO from rat aorta is elevated after incubation with M1 protein in vitro. M1 protein causes vasoplegia in rat aorta, an effect partly reversed by the NOS inhibitor, N_ω_-nitro-L-arginine methyl ester (L-NAME), which further supports involvement of M1 protein-induced NO release [12]. NOS inhibitors have, however, failed to restore hypotension in sepsis patients [17, 20]. This is in line with the finding of the present study that incubation with neither M1 protein nor LPS increased NO-production, indicating that the hyporesponsiveness to noradrenaline is mediated via mechanisms not involving NOS.

M1 protein and LPS are examples of pathogen-associated microbial patterns (PAMPs), which bind to toll-like-receptors (TLR), a receptor family with members expressed by e.g. leukocytes and endothelial cells. TLR2, TLR4 and TLR9 are present on endothelial cells and activation of these receptors by PAMPs modulates endothelial pathways involved in inflammation, permeability and coagulation via activation of intracellular MyD88 followed by downstream signaling via MAPK family members and activation of transcription factors including NF-κB [21]. One of the major NF-κB target genes is IL-6 upregulating the cytokine IL-6 [13]. In the present study we found that levels of IL-6 are increased in the medium after incubation with M1 protein. IL-6 has been used as a biomarker for sepsis since baseline plasma levels ranging between 1 and 5 pg mL^− 1^ may increase million-fold during sepsis, [22]. Furthermore, high levels of IL-6 correlate with sepsis mortality [23, 24]. Elevated IL-6 levels correlate with peripheral vasodilatation [25] as well as endothelial dysfunction [26]. The question of how IL-6 can impair endothelial cell function then rises. Endothelial cells do not express IL-6 receptors [27], but soluble IL-6 receptors (sIL-6R) are generated by neutrophils and formed IL-6/sIL-6R complexes trigger phosphorylation and redistribution of VE-cadherin leading to vascular leakage [28]. Thus, the observed M1 protein-induced rise in IL-6 may not only be regarded as a sign of general activation of innate immunity but also as a pathophysiological link between streptococcal infection and a failing circulatory system. Since the vessel segments were clear from adherent tissue and had been rinsed from visible blood remnants, it is plausible that the endothelial cells, and/or other cells of vascular wall, are responsible for the increased cytokine production.

The close relationship between M1 protein and IL-6 suggests that the dysregulated hyperinflammatory state during Streptococcal sepsis may respond to treatment with IL-6 inhibitors. IL-6-inhibitors, such as the humanized monoclonal antibody (tocilizumab), are approved for the treatment of rheumatoid arthritis [14], but beneficial effects of cytokine-blockade drugs to improve outcome in sepsis has yet to be demonstrated.

We found that the levels of IL-6 and IL-8 are increased in the medium after incubation with M1 protein but only in the presence of plasma. We further investigated if any of the main components of plasma (fibrinogen, albumin or IgG) was responsible for evoking the inflammatory response of the vascular wall to M1 protein. It was found that the cytokine levels were significantly higher in the vials with the combination M1 and fibrinogen. The mechanisms leading to inflammation and decreased effect of vasopressors by M1 protein in human omental arteries remain to be elucidated. In rodent aorta, immunohistochemistry as well as functional experiments on tissue from knock-out animals demonstrate that M1 protein in the absence of fibrinogen binds to both TLR2 and TLR4 while it activates only TLR2 [12]. In the same study it was demonstrated that M1 protein binds to TLR2 in human blood vessels but whether activation follows was not investigated. Our observation that fibrinogen enhances the effect of M1 protein points to another mode of action. It has been demonstrated that M1 protein forms complexes with fibrinogen in turn crosslinking β_2_ integrins on neutrophils resulting in degranulation and release of HBP [10]. Whether such complexes interact with vascular β_2_ integrins remains to be elucidated. In the absence of plasma, M1 protein tended to increase cytokine release in a concentration-dependent manner but in the presence of plasma, M1 protein at 1 μg mL^− 1^ was more effective than at 10 μg mL^− 1^. Interestingly, in accordance with the present findings, Herwald and colleagues found that M1 protein at 1 μg mL^− 1^ is more than twice as active in forming precipitate with plasma diluted 1:10 and in turn activates neutrophils more than M1 protein at 10 μg mL^− 1^ [10]. This confirms that some ratios between M1 protein and plasma are optimal for formation of complex and subsequent pro-inflammatory action.

Plasma levels of M1 protein during infections with Streptococcus pyogenes are unknown but immunoreactivity of M1 protein and fibrinogen have been found to co-localize within soft tissue in patients with necrotizing faciitis [10]. During severe infections, levels of fibrinogen may vary considerably from elevated levels of an acute phase reactant early on to consumption and depletion following disseminated intravascular coagulation. The present results suggest that fibrinogen substitution to patients with streptococcal sepsis should be restricted to cases with life threatening bleeding due to the potential unwanted effect of potentiating the pro-inflammaroty effect of bacterial M1 protein.

## Conclusions

In conclusion, we have shown that M1 protein, in the presence of plasma, have both an inflammatory and a vasoplegic effect on the human vascular wall. The main component in plasma responsible for this effect is fibrinogen. *Streptococcus pyogenes* expressing M1 protein are highly virulent causing severe infections. The present results suggest that by forming complexes with fibrinogen in turn stimulating cytokine release and causing vasoplegia, M1 protein contributes to the circulatory failure and septic shock connected to these conditions.

## Abbreviations

ANOVA: Analysis of variance
CBA: Cytometric bead array
FACS: Fluorescence-activated cell sorting
HBP: Heparin-binding protein
IgG: Immunoglobulin
IL: Interleukin
L-NAME: L-arginine methyl ester
MAPK: Mitogen-activated protein kinase
NO: Nitric oxide
NOS: Nitric oxide synthase
PAMP: Pathogen-associated microbial pattern
SE: Standard error of the mean
sIL-6R: Soluble IL-6 receptors
TLR: Toll-like receptor
TNF: Tumor necrosis factor
